# Destructive effect of quinone-containing compounds on cytochrome P450: Arbutin as a double-edged sword

**Published:** 2016

**Authors:** Ahmad Karkhah

**Affiliations:** 1 Student Research Committee, School of Medicine, Babol University of Medical Sciences, Babol, Iran.

**Keywords:** Cyclosporin A, Oxidative stress, Arbutin, Cytochrome P450

**Dear Editor,**

I read with great interest the article by Khadir F et al. that was published in your prestigious journal discussing about the protective effects of arbutin on lipid peroxidation in cyclosporine-treated rats (1). The authors demonstrated that arbutin has protective effect against cyclosporine A (CsA)-induced toxicity and further at dose 50 mg/kg/bw significantly neutralizes CsA-induced oxidative stress as shown by the decreased serum lipid peroxidation. Therefore, they suggested that arbutin at dose of 50 mg/kg/bw could probably inhibit the release of arachidonic acid through inhibition of phospholipase A2 and consequently decrease lipid peroxidation and free radical production. Interestingly, it has also been reported in this study that the combination of CsA and arbutin 100 mg/kg/bw increase lipid peroxidation. In fact, their results indicated that arbutin 100 mg/kg/bw increases lipid peroxidation and also induces oxidative damage. Although the findings reported in this study are worthwhile and can introduce new approaches to prevent from dose-dependent side effects of CsA such as nephrotoxicity, hypertension and hepatotoxicity, it is necessary first to recognize the exact CsA metabolism pathway and chemical compounds which may interfere with this pathway. It is well-established that arbutin is a glycosilated hydroquinone that has a variety of pharmacological activities and therapeutic properties (2). There are several lines of evidence indicating that interactions of quinone-containing compounds lead to destruction of P450 in rat and human liver microsomes (3). Quinone-containing compounds stimulate the production of reactive oxygen species which are capable to induce P450 heme destruction (4, 5). Taken together, P450 destruction by arbutin as a quinone-containing compounds may result in a major disturbance in CsA metabolism leading to a remarkable increase in serum CsA concentration which triggers its toxicity. Therefore, increased lipid peroxidation and oxidative damage of arbutin 100 mg/kg/bw might probably be related to P450 destruction by arbutin and high CsA concentration in serum. It is suggested that authors measure serum CsA concentration in arbutin-treated rats and P450 destruction along with peroxidation of lipids to determine the exact role of various concentration of arbutin in CsA metabolism and peroxidation of lipids.
